# Endophyte community shifts in *Rubus chingii* during fruit ripening are associated with key metabolites

**DOI:** 10.3389/fpls.2025.1727436

**Published:** 2025-12-19

**Authors:** Yin Xie, Di Dai, Huiting Zeng, Yingying Tian, Chao Zou, Yan Meng, Zhaoxiang Wu, Jing Li

**Affiliations:** 1 Laboratory of Traditional Chinese Medicine Development and Application, Jiangxi Provincial Institute of Traditional Chinese Medicine, Nanchang, Jiangxi, China; 2 Jiangxi Provincial Engineering Technology Research Center for Quality Standards of Traditional Chinese Medicine, Nanchang, Jiangxi, China; 3 Key Laboratory of Comprehensive Utilization of Jiangxi Traditional Chinese Medicine Resources, Nanchang, Jiangxi, China; 4 Jiangxi Research Center for the Protection and Development of Traditional Chinese Medicine Resources, Nanchang, Jiangxi, China; 5 Institute of Resources and Environment, Jiangxi Academy of Sciences, Nanchang, Jiangxi, China

**Keywords:** *Rubus chingii*, endophytic microbiome, secondary metabolites, Chinese medicinal materials, sustainable horticulture

## Abstract

**Introduction:**

The fruit of *Rubus chingii* Hu is a prized traditional medicine and functional food, with its quality predominantly determined by its secondary metabolites. While the metabolic dynamics during fruit ripening are documented, the role of the endophytic microbiome, a key regulator of plant physiology, remains entirely unexplored.

**Methods:**

An integrated approach, combining 16S/ITS amplicon sequencing with spectroscopic and chromatographic analyses, were employed to investigate the correlation between the endophytic microbiome and the metabolome across four distinct ripening stages of *R. chingii* fruit.

**Results:**

Significant stage-dependent shifts in the community structure of both bacterial and fungal endophytes were revealed in this study. Notably, Spearman correlation analysis identified specific microbial taxa, including the bacterial genera *Geodermatophilus* and *Brevundimonas*, and the fungal yeasts *Metschnikowia* and *Starmerella*, that were significantly positively correlated with the accumulation of key secondary metabolites (ellagic acid, flavonoids, and phenolic acids). Concurrently, the content of these beneficial metabolites and the fruit’s antioxidant capacity decreased markedly as ripening progressed.

**Discussion:**

This study provides the first evidence of a structured succession in the endophytic microbiome of *R. chingii* fruit and its close association with the dynamics of medically relevant metabolites. The findings propose that the ripening process is a tripartite interplay between host development, microbial succession, and metabolic reprogramming. The identified keystone taxa represent promising targets for future microbiome-based strategies to manipulate fruit quality, offering novel insights into the role of the microbiome in medicinal plant biology and its potential application in sustainable agriculture.

## 1 Introduction

The concept of “endophyte” was first conceptualized by De Bary in 1866 (De Bary, 1986). These microorganisms reside within plant tissues without causing apparent disease and can influence host growth, stress resilience, and metabolic output through diverse mechanisms (Hardoim et al., 2015; Fadiji and Babalola, 2020). A growing body of evidence suggests that the endophytic microbiome is not a passive passenger but an active contributor to plant metabolic phenotypes, forming a complex holobiont system (Vandenkoornhuyse et al., 2015; Trivedi et al., 2020). This perspective is supported by studies in medicinal plants, such as *Cinnamomum migao* H. W. Li, *Phellodendron amurense* Rupr., and *Pueraria thomsonii* Benth., where endophytes have been shown to significantly influence the accumulation of volatile oils, alkaloids, and isoflavones, respectively (Hu et al., 2025; Zhang et al., 2024; Xu et al., 2025).

The assembly of the endophytic microbiome is a dynamic process governed by ecological principles such as dispersal, selection, and species interactions (Vellend, 2010). During host development, the internal physicochemical environment-shaped by tissue morphology, nutrient availability, and defensive metabolites-acts as a strong filter, selecting for microbial colonists suited to each stage (Trivedi et al., 2020; Li et al., 2023). Understanding this dynamic assembly is crucial, as it can reveal critical windows for microbiome-mediated intervention to improve crop quality (Singh et al., 2024). In fruits, this successional process remains underexplored, particularly in non-model species with dual medicinal and dietary uses.

*Rubi Fructus*, the dry unripe fruits of *Rubus chingii* Hu, known as “Fu-Pen-Zi” in the Pharmacopoeia of the People’s Republic of China, is a clinically important medicinal fruit used to treat conditions including spermatorrhea, enuresis, and visual impairment (Pharmacopoeia Commission, 2025). Its pharmacological activity is attributed to a diverse phytochemical profile, rich in ellagic acid, flavonoids, and total phenolic compounds with demonstrated antioxidant, anti-inflammatory, and anti-osteoporotic effects (Yu et al., 2019; He et al., 2022). As a recognized medicine-food homology species, the unripe fruits are used medicinally, while ripe berries are consumed as food. Recent metabolomic studies have revealed pronounced quantitative changes in these bioactive compounds during ripening (Yang et al., 2025; Sun et al., 2021), a phenomenon also observed in other fleshy fruits such as blueberries and tomatoes, where metabolic shifts are linked to both ecological and quality attributes (Klee and Tieman, 2018; Zhang M. Q. et al., 2023). Despite these phytochemical insights, the role of the endophytic microbiome in *R. chingii* fruit development and metabolite accumulation remains largely unknown. Addressing this gap requires an integrated eco-metabolomic framework that simultaneously tracks microbial succession and metabolome restructuring-an approach successfully applied in other crop systems (Li et al., 2024).

In this paper, we applied a multi-omics approach, specifically integrating microbiome (16S rRNA gene sequencing) and metabolomics (liquid chromatography-mass spectrometry) data. We specifically targeted three primary metabolites (polysaccharides, flavonoids, phenolic acids) and five secondary metabolites (ellagic acid, rutin, kaempferol-3-O-rutinoside, quercitrin, and tilianin) for quantification. These compounds were selected because they are recognized as the principal bioactive constituents contributing to the antioxidant and medicinal properties of *R. chingii* fruit, and their dynamics across ripening stages are pivotal for understanding quality formation (Li X. B. et al., 2021; He et al., 2022; Yang et al., 2025). We hypothesized that the ripening of *R. chingii* fruit involves a structured ecological succession of the endophytic microbiome that is intrinsically linked to the characteristic reprogramming of the host metabolome. Our findings aim to elucidate the ecological functions of endophytes in *R. chingii*, providing a novel microbiological perspective on fruit quality formation.

## 2 Materials and methods

### 2.1 Sample collection

The fruits were collected from nine healthy, three-year-old *R. chingii* Hu plants of the “Daguo” cultivar, grown in the well-drained, sandy loam soil at the Traditional Chinese Medicine Popular Science Base in Yongxiu County. They were classified into four distinct ripening stages based on color and maturity: light green (initial stage, RYL), green (RYG), yellow (RYY), and red (post-ripening, RYR) stages. The four stages were harvested at 30, 40, 50, 60 days post-anthesis, respectively. Fruits from every three plants were pooled to form one biological replicate, resulting in a total of 12 samples (3 replicates × 4 stages). All samples were aseptically collected in sterile plastic bags, immediately placed on dry ice, and processed within 24 hours. Each sample was divided into two aliquots: one for endophyte analysis and the other for metabolite profiling. For endophyte analysis, the samples underwent a rigorous surface sterilization procedure. They were first rinsed with tap water, followed by three washes with sterile distilled water. Subsequent sterilization was carried out by immersion in 75% ethanol for 1 min and 5% sodium hypochlorite solution for 2 min, followed by three final rinses with sterile water. The effectiveness of the sterilization was verified by inoculating the final rinse water onto both NA medium (incubated at 37°C for 3 days) and PDA medium (incubated at 28°C for 5 days) to confirm the absence of microbial growth. The surface-sterilized fruits were then stored at -80°C until further analysis. For metabolite analysis, the corresponding aliquot was oven-dried at 40°C, finely ground into powered, and prepared for subsequent profiling. The entire surface sterilization and sample processing protocol followed established methodologies to minimize epiphytic contamination and preserve the integrity of endogenous metabolites (Lima et al., 2021).

### 2.2 Analysis of agronomic traits and metabolite profiles

The length and width of fresh fruits were measured using a vernier caliper (accuracy: 0.01 mm). Individual fruit fresh weight was determined with an analytical balance, while sugar content (Brix, %) and titratable acidity (%) were assessed using a digital sugar meter (PAL-BX, Atago, Japan).

Based on previous research, three primary metabolites-polysaccharides, flavonoids, and total phenols-were quantified. Polysaccharide content was determined by the phenol-sulfuric acid method, total phenolic content via the Folin-Ciocalteu assay (Li X, et al., 2021), and total flavonoid content using an aluminum chloride colorimetric method on a UV-Vis spectrophotometer. The latter method is based on the formation of a stable complex between aluminum ions and the keto group of flavonoids (Zhishen et al., 1999; Kumar and Pandey, 2013). The DPPH free radical scavenging assay was employed to evaluate the antioxidant activity (Tian et al., 2025; Munteanu and Apetrei, 2021). To quantify the potency, the half-maximal effective concentration (EC_50_) was determined from the scavenging curve as the sample concentration required to achieve 50% radical scavenging. All spectrophotometric analyses followed well-established plant biochemistry protocols.

Based on previous studies (Li Y, et al., 2021; He et al., 2022; Yang et al., 2025), five secondary metabolites: ellagic acid, rutin, kaempferol-3-O-rutinoside, quercitrin, and tilianin, were selected as target analytes. Fruit samples were ground into powder and sieved through a 300 μm mesh. A precisely weighed aliquot (0.5 g) of the powder was extracted with 20 mL of 70% (v/v) methanol in an ultrasonic bath (300 W, 60 kHz) at 60°C for 30 min. After cooling, the extract was made up to the original weight with solvent and filtered through a 0.22 μm membrane prior to HPLC analysis. Chromatographic separation was performed on an Agilent Extend C18 column (250 mm × 2.1 mm, 4.5 μm) using a gradient elution program with (A) acetonitrile and (B) 0.1% (v/v) aqueous formic acid as the mobile phase. The gradient was set as follows: 0–10 min, 10% A; 10–16 min, 10%-15% A; 16–40 min, 15%-18% A; 40–45 min, 18%-24% A; 45–60 min, 30% A. The detection wavelength, flow rate, column temperature, and injection volume were 360 nm, 1.0 mL/min, 35°C, and 20 μL, respectively. Individual stock solutions of reference standards were prepared in methanol at specified concentrations: ellagic acid (5.265 mg/mL), kaempferol-3-O-rutinoside (1.83 mg/mL), rutin (0.916 mg/mL), quercitrin (0.866 mg/mL), and tilianin (0.820 mg/mL). Calibration curves were established by analyzing serially diluted standard solutions under the same chromatographic conditions. Compound identification was based on retention time matching with authentic standards, and quantification was carried out using the corresponding calibration curves.

### 2.3 DNA extraction, high-throughput sequencing, and bioinformatic analysis

Total genomic DNA was extracted from surface-sterilized fruit samples using the FastPure Soil DNA Isolation Kit (Magnetic Bead) (MJYH, Shanghai, China) according to the manufacturer’s protocol. DNA quality and concentration were assessed by 2.0% agarose gel electrophoresis and a NanoDrop 2000 spectrophotometer (Thermo Scientific, USA), respectively. Qualified DNA samples were stored at -80°C until further use. Bacterial 16S rDNA amplification was performed in two sequential PCR steps. The first step used primers 799F (5’-AACMGGATTAGATACCCKG-3’)/1392R (5’-ACGGGCGGTGTGTRC-3’), and the second step used primers 799F (5’-AACMGGATTAGATACCCKG -3’)/1193R (5’-ACGTCATCCCCACCTTCC-3’). Each 20 μL PCR reaction contained 10 μL of 2× ProTaq HS PCR Master Mix, 0.8 μL each of forward and reverse primer (5 μM), 10 ng template DNA, and ddH_2_O to volume. Amplification was conducted on a T100 Thermal Cycler (Bio-Rad, USA) under the following program: initial denaturation at 95°C for 3 min; 27 cycles of 95°C for 30 s, 55°C for 30 s, and 72°C for 45 s; followed by a final extension at 72°C for 10 min. The second PCR used identical conditions but was run for 13 cycles. Fungal ITS regions were amplified in a single step using primers ITS1F (5’-CTTGGTCATTTAGAGGAAGTAA-3’)/ITS2R(5’-GCTGCGTTCTTCATCGATGC-3’) with 35 cycles under the same thermal profile. All reactions were performed in three technical replicates. PCR products were purified with a PCR Clean-Up Kit (YuHua, Shanghai, China) and quantified using a Qubit 4.0 fluorometer (Thermo Fisher Scientific, USA). Equimolar amounts of amplicons were pooled and sequenced on an Illumina NextSeq 2000 platform (Illumina, USA) in paired-end mode by Majorbio Bio-Pharm Technology Co., Ltd. (Shanghai, China). Raw sequencing data were deposited in the NCBI Sequence Read Archive under accession number PRJNA1303412.

Bioinformatic processing was performed in QIIME2. After demultiplexing, sequences were quality-filtered, denoised, and merged to generate amplicon sequence variants (ASVs). To obtain a high-quality dataset for ecological analysis, several filtering steps were applied: (1) ASVs classified as Chloroplast or Mitochondria were removed from the bacterial and fungal datasets, respectively. (2) ASVs that could not be classified to any kingdom were removed. (3) To minimize the influence of potential sequencing artifacts, ASVs with a total abundance of less than 2 (i.e., singletons and doubletons) were filtered out from all samples. All subsequent analyses, including diversity metrics, taxonomic composition, and correlation analysis, were performed on this filtered dataset. Bacterial and fungal amplicon sequence variants (ASVs) were clustered at 97% similarity, corresponding to conventional species-level resolution (Schloss et al., 2009). Rarefaction curves were generated to evaluate sequencing depth, and alpha diversity indices were calculated using Mothur (version 1.30.2). Functional potential of bacterial communities was predicted using PICRUSt2 (http://picrust.github.io/picrust/), while fungal functional guilds were inferred with FUNGuild (http://www.funguild.org/). The overall analytical pipeline follows current best practices in microbial ecology (Bolyen et al., 2019). Associations between host metabolites and endophytic diversity/abundance were evaluated using Spearman’s rank correlation analysis.

### 2.4 Data analysis

The normality of all data (agronomic traits, metabolite concentrations, and microbial diversity indices) was assessed using the Shapiro-Wilk test. Homogeneity of variances was verified using Levene’s test. Data that met the assumptions for parametric tests were analyzed by one-way ANOVA followed by Tukey’s HSD *post-hoc* test in SPSS Statistics 26.0 (IBM Corp., USA). Data that met the assumptions for parametric tests were analyzed by one-way ANOVA followed by Tukey’s HSD *post-hoc* test; otherwise, the non-parametric Kruskal-Wallis test with Dunn’s *post-hoc* test was applied. All figures were prepared using Origin 2021 (OriginLab Corp., USA). The polysaccharides and flavonoids contents were performed by UV-2600i (Shimadzu Corp., Japan) and the DPPH free radical scavenging assay was conducted by Microplate Reader Readmax 1900 (Shanghai Shanpu Bio-Technology Co., Ltd., China). The concentrations of secondary metabolites were determined by High-Performance Liquid Chromatography (Waters 2695, USA) in the software of empower 3.0 for data acquisition and initial processing. 16S and ITS sequencing data were processed in QIIME2 (version 2023.9). Microbial community analyses, including non-metric multidimensional scaling (NMDS) and heatmap visualization, were conducted in R (version 3.3.1) with the vegan and pheatmap (v1.0.8) packages, respectively. Additional analyses were performed using the boot (version 1.3.18) and stats (version 3.3.1) packages for statistical computing, while functional prediction of microbial communities was carried out with Tax4Fun (version 0.3.1).

## 3 Results

### 3.1 Dynamic changes in agronomic traits and metabolite profiles during fruit development

**Figure 1:**
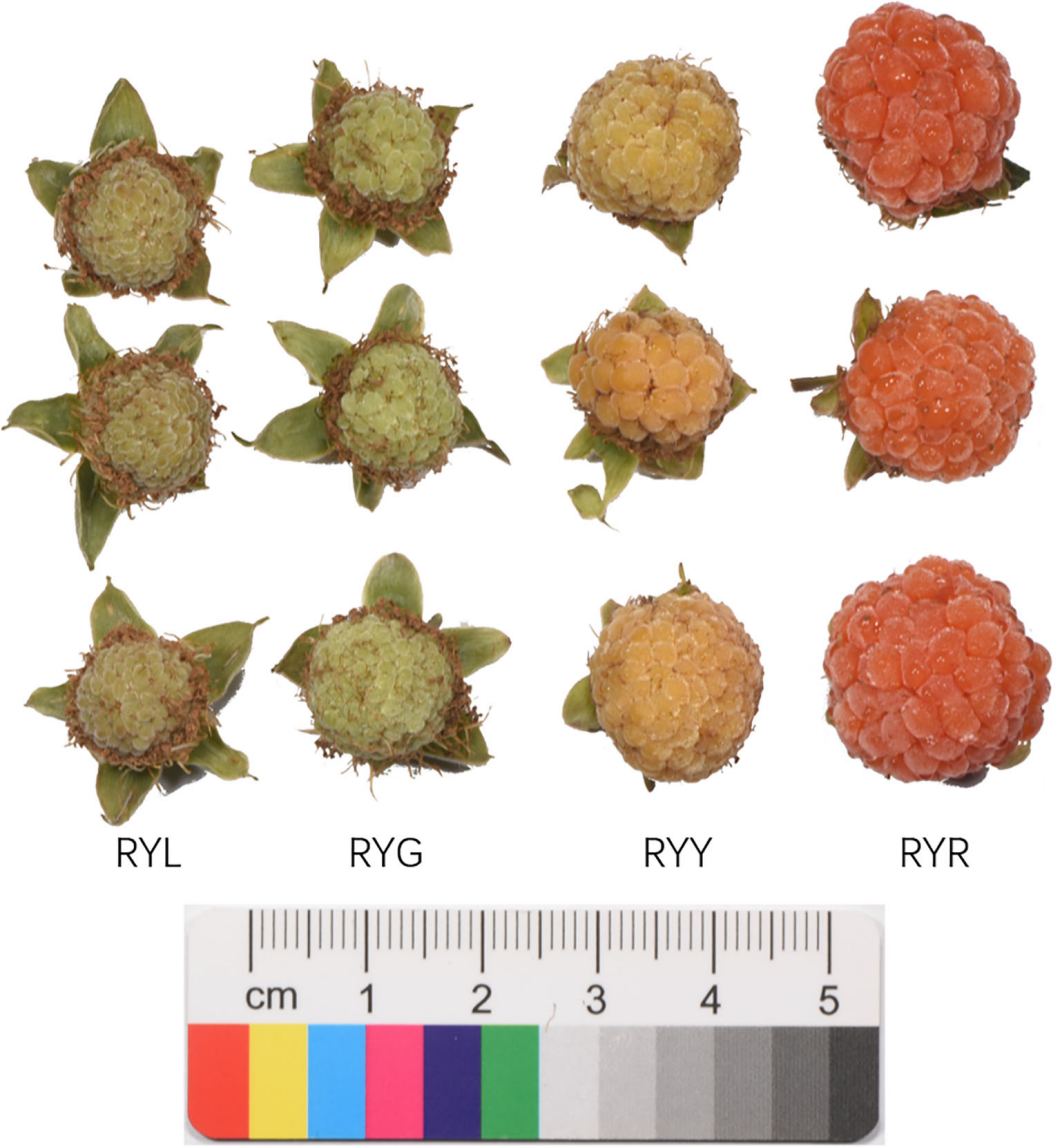
Appearance and color of *R. chingii* fruits from four harvest stages. RYL (light green), RYG (green), RYY (yellow), RYR (red).

Morphometric analyses revealed a continuous increase in fruit size throughout the development of *R. chingii*, as evidenced by the progressive enlargement in both length and width (**Figure 1**). Concurrent with color deepening, notable shifts in flavor attributes were observed. The soluble solids contents exhibited a gradual increase, whereas titratable acidity showed an initial rise followed by a decline during later stages. Furthermore, *in vitro* antioxidant assessment via the DPPH radical scavenging assay indicated a progressive decline in antioxidant capacity with advancing fruit maturation (**Table 1**).

**Table 1:** Agronomic traits and flavor of *Rubus chingii* fruits from four harvest stages.

Sample	Agronomic traits			Flavor		DPPH
	Length (mm)	Width (mm)	Fresh weight (g)	Brix (%)	Acidity (%)	EC_50_ (μg/mL)
RYL	10.95 ± 0.37b	10.15 ± 0.19b	0.69 ± 0.03b	7.57 ± 0.30b	1.08 ± 0.02b	318.46 ± 2.34d
RYG	11.79 ± 0.16b	11.28 ± 0.27b	0.84 ± 0.03b	8.30 ± 0.45b	2.01 ± 0.06a	345.49 ± 18.24c
RYY	12.48 ± 0.98b	12.12 ± 0.53b	1.07 ± 0.21b	7.97 ± 0.77b	2.12 ± 0.05a	573.39 ± 63.84b
RYR	17.31 ± 2.18a	16.17 ± 1.39a	2.50 ± 0.78a	12.73 ± 0.83a	0.96 ± 0.28c	2613.00 ± 156.60a

Different letters after the data indicate a significant difference level of 5%.

**Figure 2:**
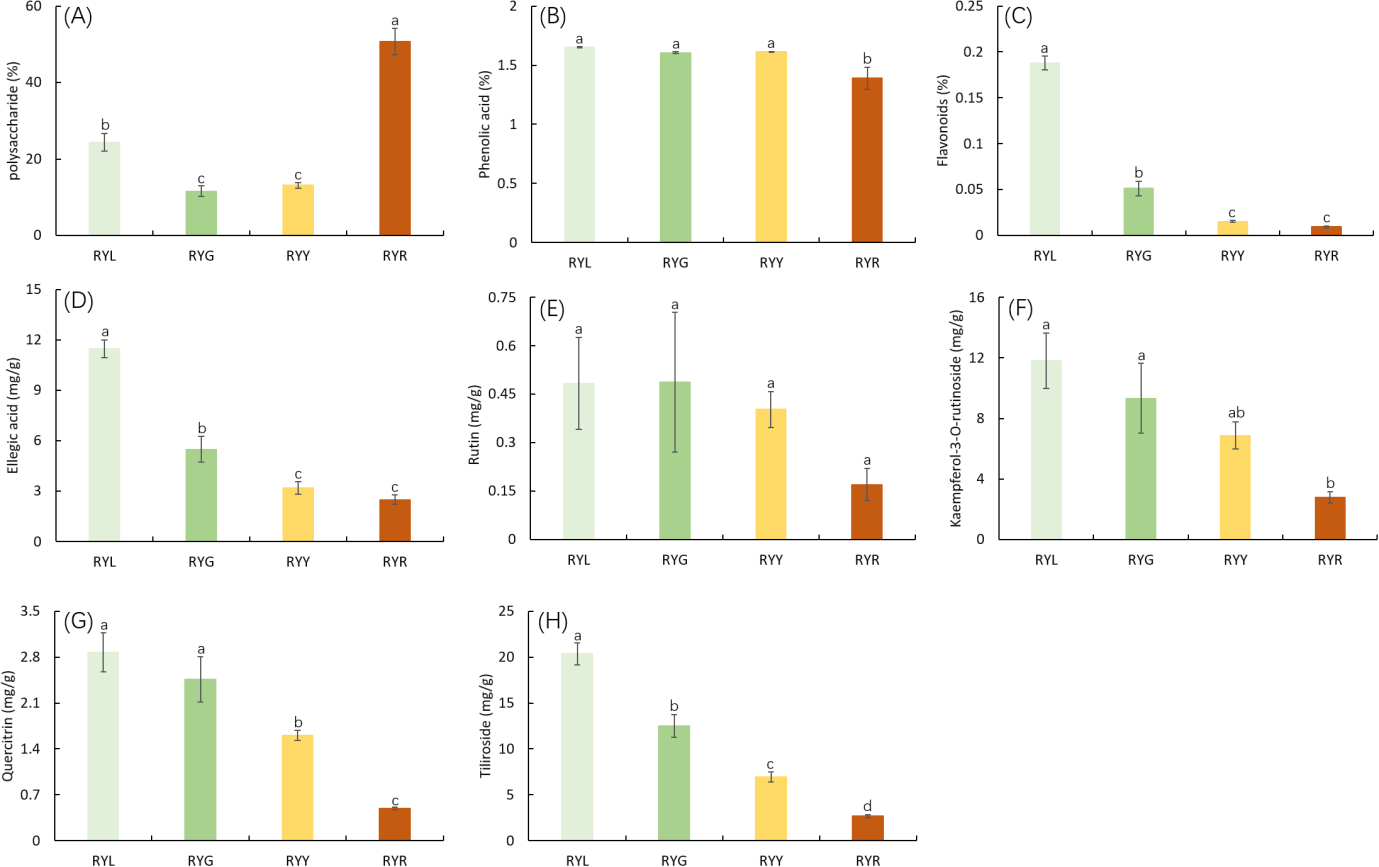
Concentrations (%, DW) of the primary metabolites (polysaccharide **(A)**, phenolic acid **(B)** and flavonoids **(C)**) and concentrations (mg/g, DW) of secondary metabolites (ellagic acid **(D)**, rutin **(E)**, kaempferol-3-O-rutinoside **(F)**, quercitrin **(G)** and tiliroside **(H)**) in *R. chingii* fruits from four harvest stages. Data are shown as mean & plusmn SD (n ≥ 3). RYL (light green), RYG (green), RYY (yellow), RVR (red). Different letters above bars indicate significant differences (P < 0.05).

Analysis of primary metabolites revealed dynamic changes during fruit development. Polysaccharide content decreased initially, then increased, reaching its peak at the red (post-ripening) stage (RYR; **Figure 2A**). Total phenol concentration remained relatively stable during the early developmental phases but decreased significantly at the RYR stage (**Figure 2B**). Total flavonoid content declined markedly from 0.19% to 0.01% as fruit color transitioned from light green to red, with no significant difference observed between the last two stages (RYY and RYR; **Figure 2C**). The five quantified secondary metabolites exhibited distinct accumulation patterns during maturation. The concentrations of ellagic acid, quercitrin, kaempferol-3-O-rutinoside, and tiliroside decreased progressively throughout fruit maturation (**Figures 2D, F, G, H**). Specifically, the concentration of ellagic acid declined from 12.5 ± 0.8 mg/g DW at the RYL stage to 2.7 ± 0.3 mg/g DW at the RYR stage. Similar significant decreasing trends were observed for the other metabolites. In contrast, rutin content remained relatively consistent during the initial developmental stages but declined noticeably from the RYY to RYR stage, although this change was not statistically significant (**Figures 2D-H**).

### 3.2 Endophytic microbial diversity

High-throughput sequencing of endophytic bacteria and fungi was performed across four ripening stages of *R. chingii* fruits, encompassing a total of 12 samples. After quality control and filtering, 366,976 high-quality bacterial 16S rRNA sequences (average length: 393.58 bp) and 1,059,733 fungal ITS sequences (average length: 167.50 bp) were retained. A total of 4,055 bacterial and 1,029 fungal amplicon sequence variants (ASVs) were identified after clustering at 97% similarity. Rarefaction curves demonstrated that ASV richness increased with sequencing depth and approached saturation in all samples, indicating adequate sequencing coverage (**Supplementary Figure S1**).

Analysis of α-diversity revealed distinct dynamics between bacterial and fungal communities (**Table 2**). Based on the average observed ASVs and Chao index, the richness of the bacterial community initially increased and subsequently decreased during fruit maturation, with the lowest values observed at the RYL stage. In contrast, fungal diversity showed an opposite trend, peaking at the RYR stage. The coverage indices for both bacterial and fungal communities exceeded 99.9%, confirming that the sequencing depth sufficiently captured the microbial diversity. These results collectively demonstrate the high reliability of the captured endophytic microbial community structure and support the feasibility of comparing microbiome variations across the four developmental stages of *R. chingii* fruits.

**Table 2:** Alpha diversity of endophytic bacteria and fungi in *R. chingii* fruits from four harvest stages.

Group	Sample	ACE	Chao	Shannon	Simpson	Coverage
Bacteria	RYL	235.07 ± 8.11b	235.29 ± 8.20b	4.60 ± 0.12b	0.02 ± 0.00b	0.99
	RYG	157.71 ± 3.36b	157.76 ± 2.95b	3.61 ± 0.23c	0.07 ± 0.03a	1.00
	RYY	761.23 ± 168.41a	754.97 ± 163.04a	5.58 ± 0.24a	0.01 ± 0.00b	0.99
	RYR	364.62 ± 14.76b	359.04 ± 12.74b	4.44 ± 0.17b	0.03 ± 0.01ab	1.00
Fungi	RYL	111.65 ± 26.54d	111.39 ± 26.56d	2.10 ± 0.38a	0.23 ± 0.08a	1.00
	RYG	113.36 ± 35.03c	116.06 ± 33.71c	2.00 ± 0.34a	0.24 ± 0.05a	1.00
	RYY	134.06 ± 2.14b	134.00 ± 2.08b	1.64 ± 0.09b	0.40 ± 0.04a	1.00
	RYR	310.95 ± 59.60a	310.75 ± 59.60a	2.96 ± 0.45a	0.16 ± 0.07b	1.00

Different letters after the data indicate a significant difference level of 5%.

### 3.3 Composition and dynamics of the endophytic microbial community

**Figure 3:**
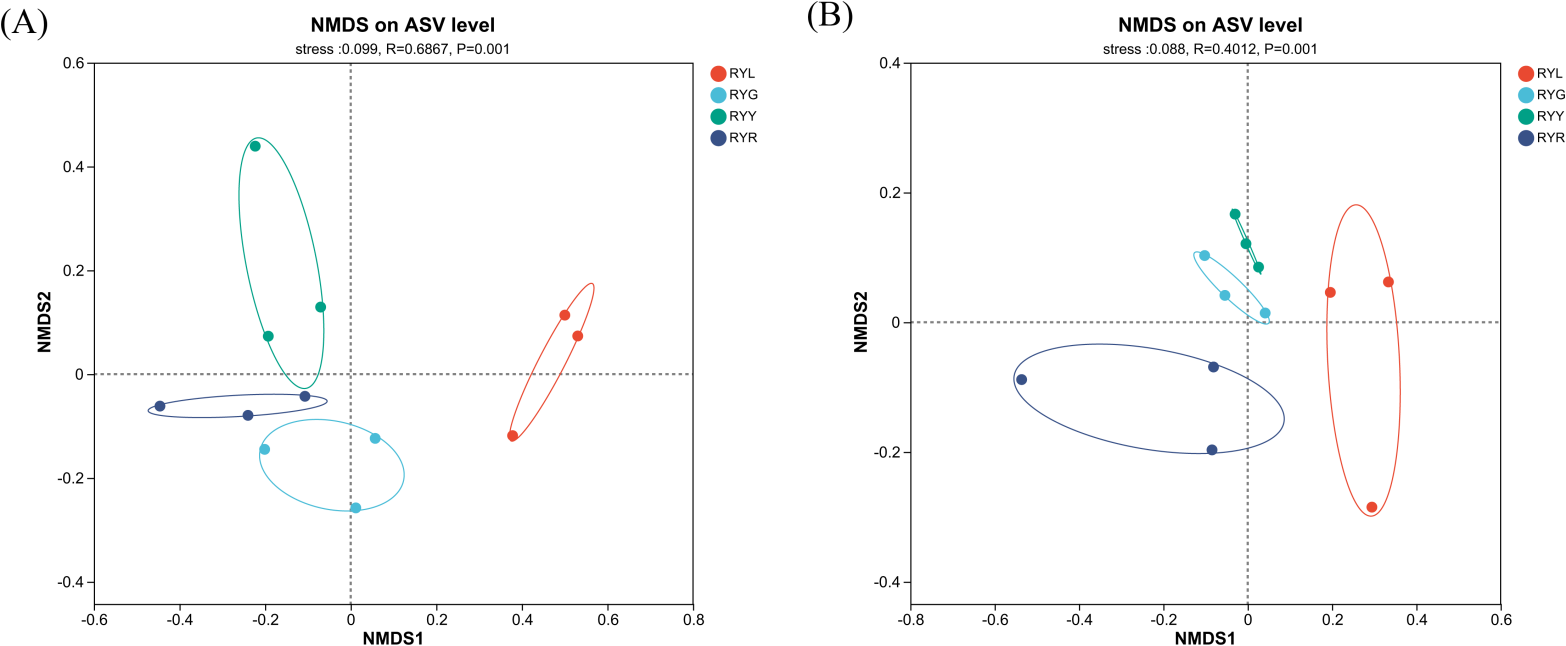
Non-metric multidimensional scaling (NMDS) analysis for bacteria **(A)** and fungi **(B)** in *R. chingii* fruit from four harvest stages. RYL (light green), RYG (green), RYY (yellow), RYR (red). Ellipses denote 95% confidence intervals.

Non-metric multidimensional scaling (NMDS) based on ASVs revealed clear shifts in the β-diversity of both bacterial and fungal communities across fruit developmental stages (**Figure 3**). PERMANOVA confirmed significant compositional differences among stages (*P* = 0.001), with stress values of 0.099 and 0.088 for bacterial and fungal datasets, respectively. The NMDS ordination, supplemented with 95% confidence ellipses, illustrated distinct clustering patterns: RYL samples formed a separate cluster, while RYG, RYY, and RYR samples grouped closely together. The auxiliary box-and-whisker plots at the top and right provided insights into data dispersion and central tendency along the NMDS1/NMDS2 axes, further supporting the observed community differences. For instance, the light blue box-and-whisker plot could directly show the distribution of samples within the RYR group. Combined with the significant difference from PERMANOVA, it could be seen that during *R. chingii* fruit ripening, the structures of bacterial and fungal communities changed significantly with the transformation of color phenotypes and metabolic substances. Thus, the NMDS ordination, combined with box-and-whisker plots and PERMANOVA, clearly revealed the differences in the structures of bacterial and fungal communities in *R. chingii* fruits from four harvest stages, providing support for elucidating the association between *R. chingii* fruits ripening and the endophytes.

**Figure 4:**
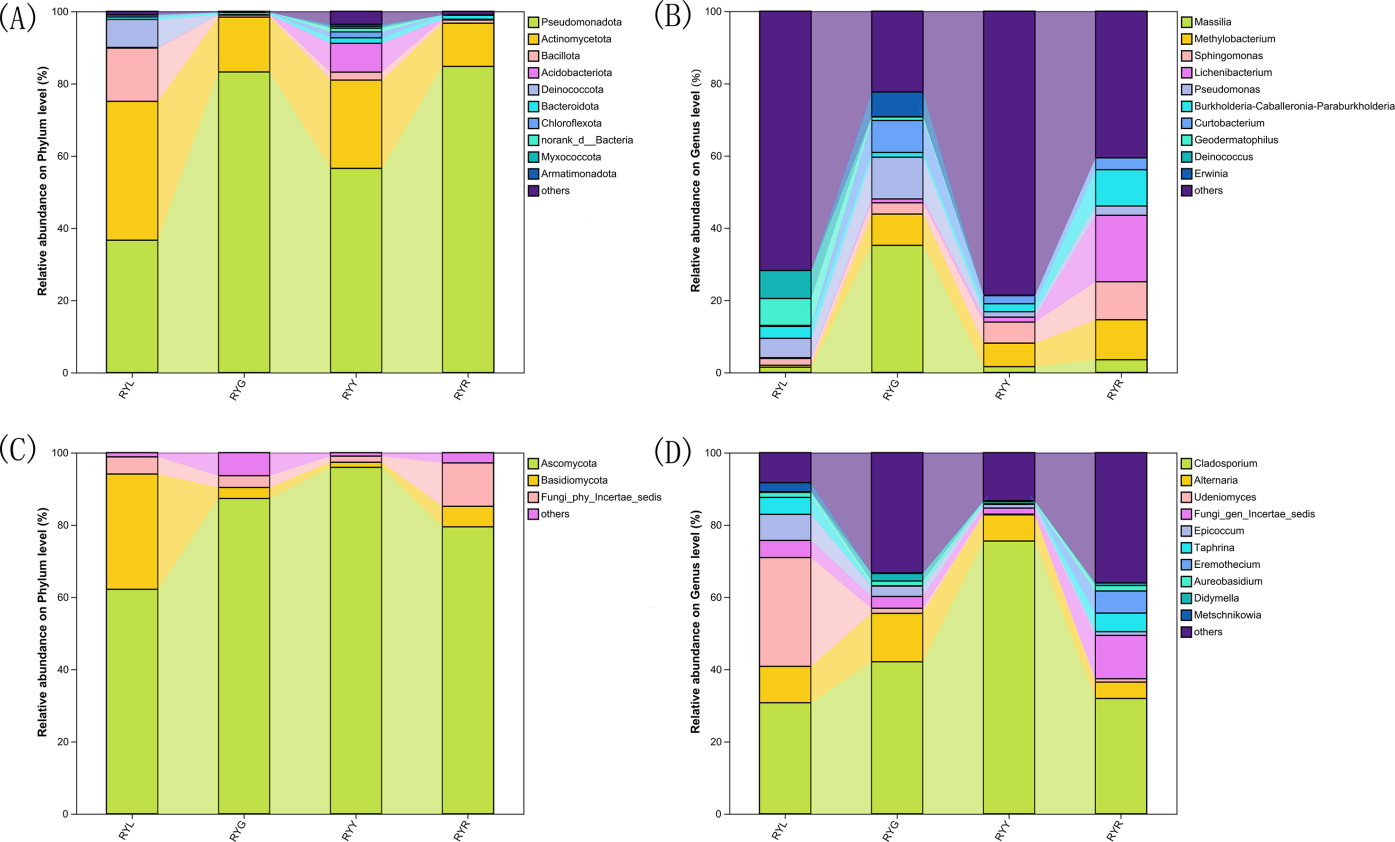
Relative abundance of bacteria **(A, B)** and fungi **(C, D)** in *R. chingii* fruits at phylum level **(A, C)** and genus level (B, D). RYL (light green), RYG (green), RYY (yellow), RYR (red).

Taxonomic assignment of ASVs (97% similarity threshold) identified 30 bacterial phyla encompassing 530 genera. The bacterial community was predominantly composed of Pseudomonadota (32.28-83.92%), Actinomycetota (4.72-56.54%) and Bacillota (0.18-21.85%). Notably, Actinomycetota was the most abundant phylum in RYL, whereas Pseudomonadota dominated in RYG, RYY, and RYR. Compared to other column of RYG, RYY and RYR, the proportions of Bacillota and Deinococcota were particularly prominent in the column chart of RYL (**Figure 4A**). In contrast, the fungal community comprised 4 phyla and 325 genera, largely dominated by Ascomycota (62.15-95.93%) and Basidiomycota (1.41-5.69%), with the most abundance of Ascomycota across all the four stages (**Figure 4C**).

**Figure 5:**
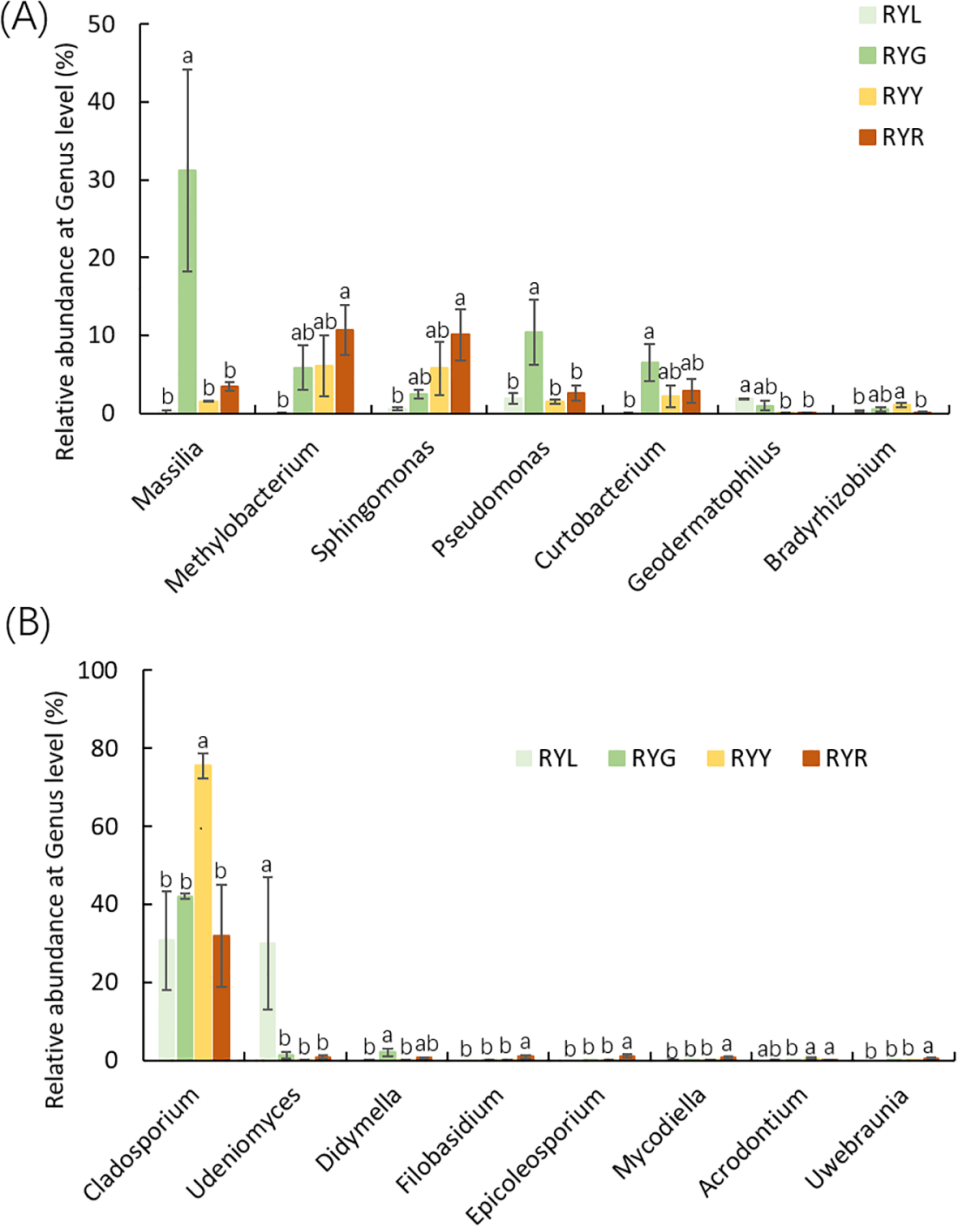
Relative abundance of bacteria **(A)** and fungi **(B)** at genus level. Different letters after the data indicate a significant difference level of 5%.

**Figure 6:**
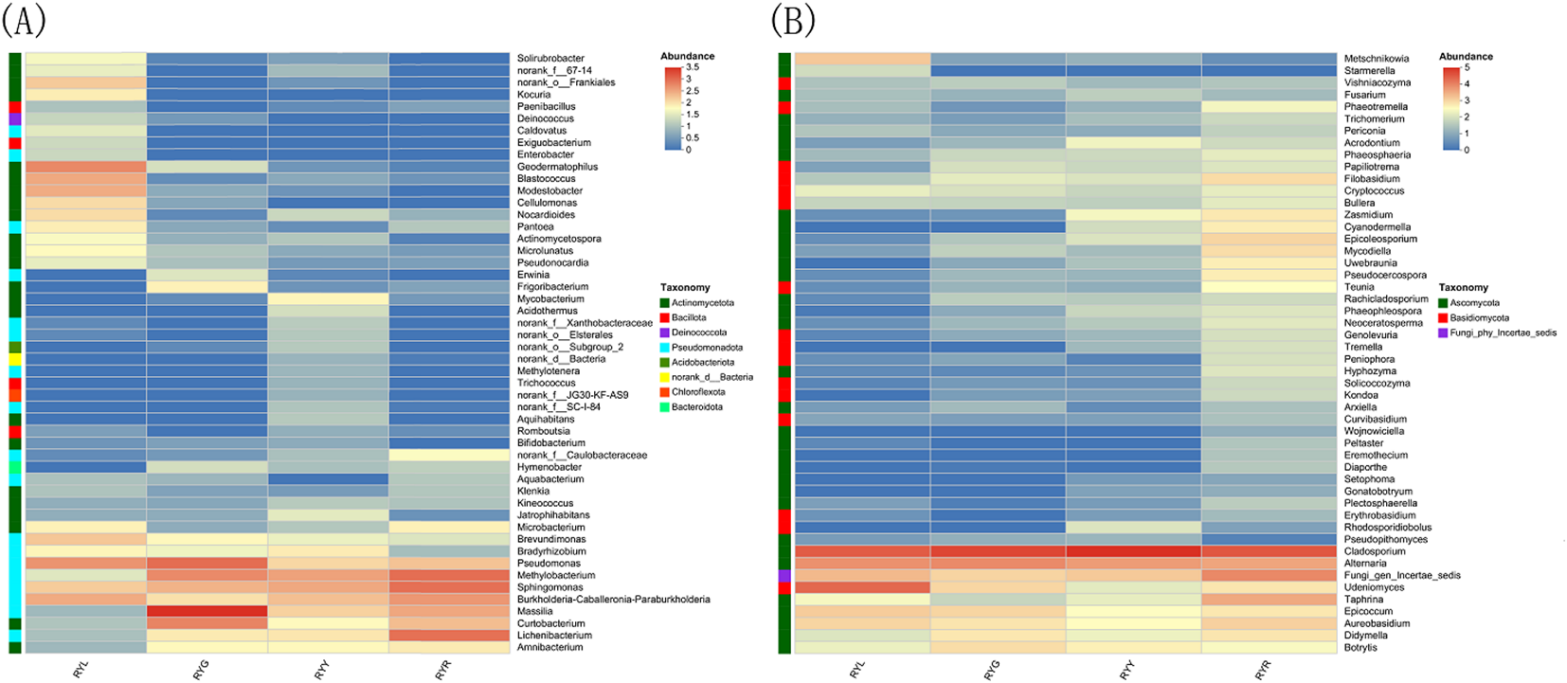
Heat map of bacteria **(A)** and fungal **(B)** communities in *R. chingii* fruits at genus level.

Statistical analysis of the relative abundance of endophytic bacteria at the genus level showed variation in samples from different fruit stages (**Figure 4B**). Several bacterial genera exhibited stage-specific high-abundance: *Massilia*, *Pseudomonas*, and *Curtobacterium* were most abundant in RYG (P < 0.05), while *Methylobacterium*, *Sphingomonas* and *Lichenibacterium* increased significantly throughout ripening, peaking in RYR. Notablely, *Sphingomonas* showed a climbing trend (**Figure 5A**). Fungal composition was predominated by *Cladosporium* across all stages (30.73–75.53%; **Figures 4D**, **6B**), with the highest relative abundance observed in RYY. *Didymella* was most abundant in RYG, while *Udeniomyces* peaked in RYL and declined thereafter. Several genera, including *Fungi_gen_Incertae_sedis*, *Epicoccum*, *Taphrina* and *Eremothecium* were significantly more abundance in RYR (P<0.05) compared to earlier stages (**Figure 5B**).

### 3.4 Correlations between endophytic microbial communities and fruit metabolites

**Figure 7:**
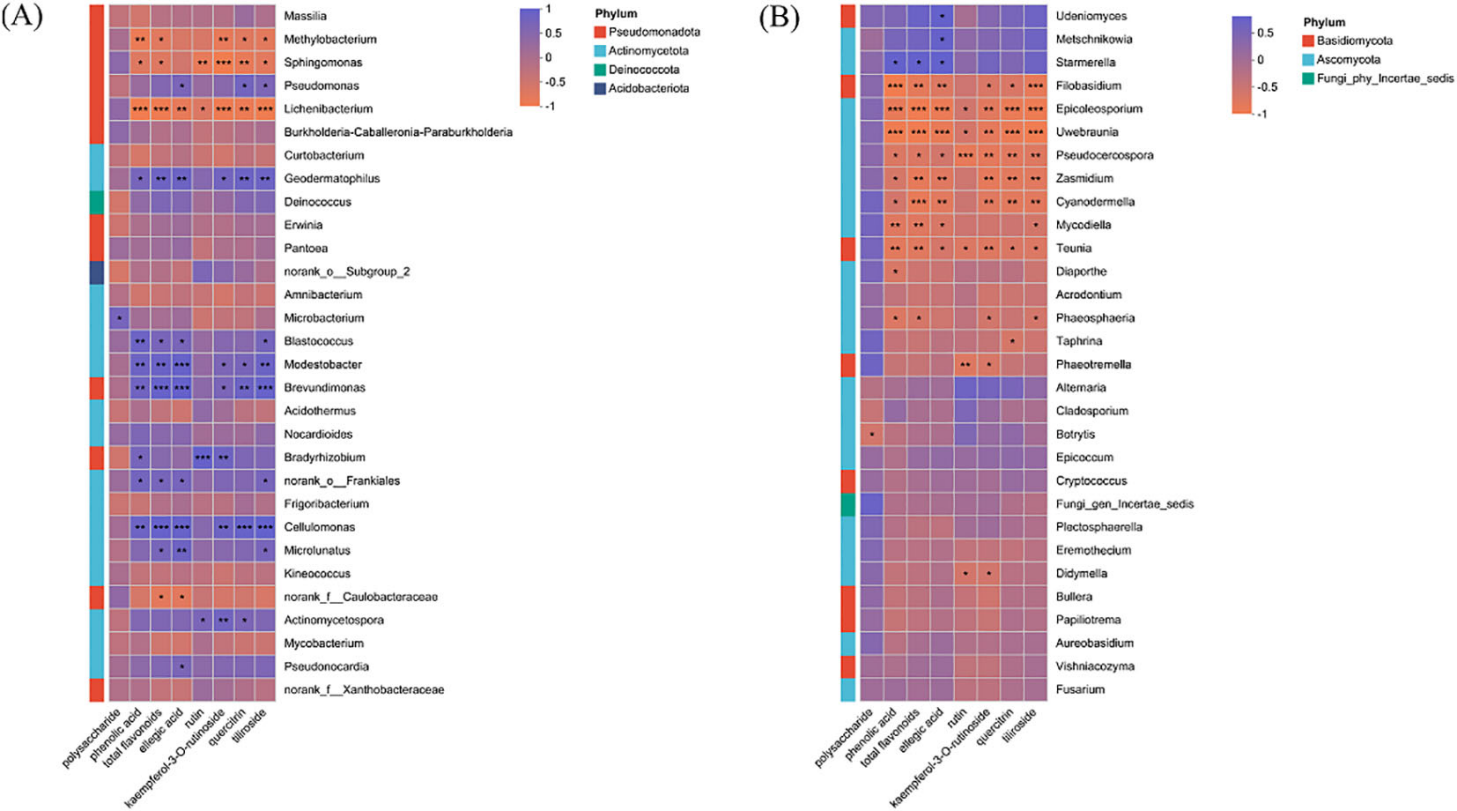
Relationship between microbial community composition at genus level and metabolites in *R. chingii* fruit. **(A)** bacteria. **(B)** fungi. ***, *P*<0.001. **, *P*<0.01. *, *P*<0.05.

Spearman correlation analysis visualized via heatmaps revealed distinct association patterns between endophytic microbial genera (bacteria and fungi) and the eight measured metabolites (**Figure 7**). Among bacterial genera (**Figure 7A**), *Microbacterium* abundance was significantly positively correlated with total polysaccharide content. *Bradyrhizobium* and *Actinomycetospora* both showed a positive correlation with rutin, kaempferol-3-O-rutinoside, quercitrin, and tiliroside. Notably, *Brevundimonas, Modestobacter*, *Geodermatophilus* and *Cellulomonas* exhibited positive correlations with multiple metabolites, including phenolic acid, flavonoids, ellagic acid, kaempferol-3-O-rutinoside, quercitrin, and tiliroside. *Pseudomonas*, *Microlunatus* and *Pseudonocardia* both showed positive correlation with ellagic acid content. In the fungal community (**Figure 7B**), *Udeniomyces* and *Metschnikowia* were significantly positively correlated with ellagic acid content. *Starmerella* was positively associated with total polyphenols, flavonoids, and ellagic acid.

### 3.5 Functional prediction of the microbial community

**Figure 8:**
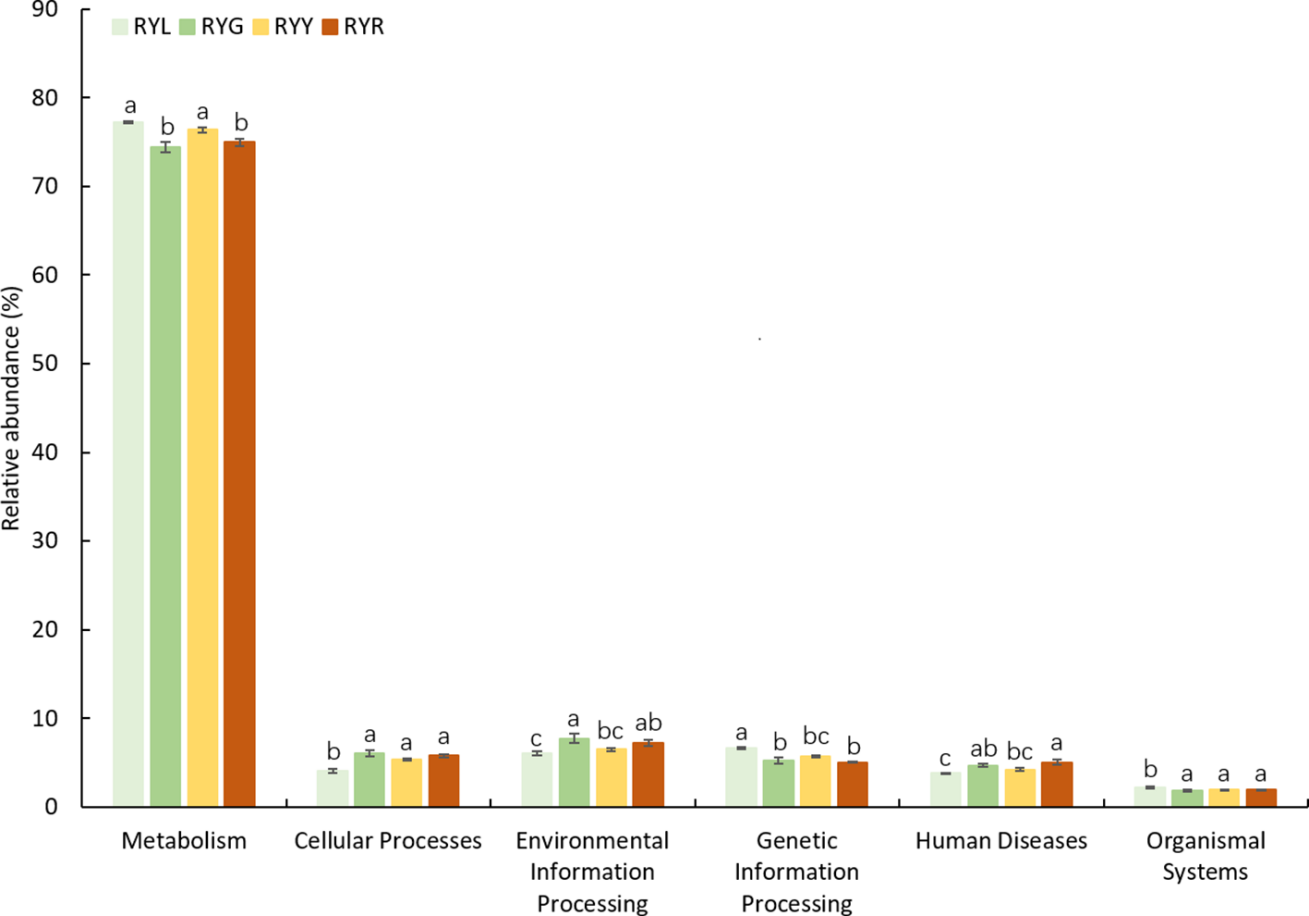
Predicted function of the bacterial community found in *R. chingii* fruits based on KEGG pathway level 1 with significant difference among the four harvest stages. Different letters after the data indicate a significant difference level of 5%.

Functional prediction of the bacterial community using PICRUSt2 identified 6 level-1 and 12 level-2 KEGG Orthology (KO) groups (**Table 3**). At level-1, metabolic processes constituted the predominant functional category (73.45-77.46%), followed by cellular processes, environmental information processing, genetic information processing, human diseases, and organismal systems; all functional categories exhibited variations across the four developmental stages (**Figure 8**). At level-2, “global and overview maps” was the most abundant category, followed by “amino acid metabolism” and “carbohydrate metabolism”; however, no significant differences were observed among stages at this level (**Supplementary Figure S2**). Based on FUNGuild annotation, fungal communities were classified into eight trophic modes (**Supplementary Figure S3**). Statistical analysis revealed that only “Saprotroph-Pathotroph-Symbiotroph” and “Saprotroph” differed significantly across fruit developmental stages.

**Table 3:** Predicted function of the bacterial community found in *R. chingii* fruits based on the KEGG pathway level 3 with significant difference among the four harvest stages.

Pathway		Relative abundance (%)			
Level 2	Level 3	RYL	RYG	RYY	RYR
Amino acid metabolism	Alanine, aspartate and glutamate metabolism	0.81 + 0.01a	0.71 + 0.01b	0.69 + 0.01bc	0.67 + 0.01c
	Arginine biosynthesis	0.50 + 0.01a	0.46 + 0.00b	0.44 + 0.01b	0.44 + 0.01b
	Cysteine and methionine metabolism	0.89 + 0.01a	0.81 + 0.02b	0.81 + 0.01b	0.8 + 0.01b
	Glycine, serine and threonine metabolism	0.84 + 0.02bc	0.87 + 0.01c	0.93 + 0.01a	0.89 + 0.02ab
	Lysine biosynthesis	0.39 + 0.01ab	0.38 + 0.00bc	0.40 + 0.01a	0.37 + 0.01c
	Lysine degradation	0.24 + 0.04b	0.40 + 0.04a	0.45 + 0.00a	0.43 + 0.02a
	Phenylalanine metabolism	0.31 + 0.03b	0.54 + 0.08a	0.53 + 0.01a	0.52 + 0.04a
	Phenylalanine, tyrosine and tryptophan biosynthesis	0.64 + 0.01a	0.56 + 0.02b	0.57 + 0.02b	0.53 + 0.01b
	Tryptophan metabolism	0.29 + 0.04a	0.45 + 0.04b	0.54 + 0.01b	0.53 + 0.02b
	Valine, leucine and isoleucine biosynthesis	0.48 + 0.02a	0.45 + 0.01ab	0.43 + 0.01bc	0.39 + 0.00c
	Valine, leucine and isoleucine degradation	0.41 + 0.06b	0.64 + 0.06a	0.77 + 0.00a	0.72 + 0.03a
Biosynthesis of other secondary metabolites	Acarbose and validamycin biosynthesis	0.07 + 0ab	0.08 + 0.01a	0.06 + 0ab	0.05 + 0b
	Carbapenem biosynthesis	0.09 + 0.01a	0.05 + 0.01b	0.05 + 0.00b	0.05 + 0.00b
	Glucosinolate biosynthesis	0.07 + 0.00a	0.07 + 0.00a	0.07 + 0.00a	0.06 + 0.00b
	Isoquinoline alkaloid biosynthesis	0.02 + 0.01b	0.06 + 0.01a	0.05 + 0a	0.06 + 0a
	Monobactam biosynthesis	0.20 + 0.00b	0.20 + 0.00b	0.23 + 0.01a	0.21 + 0.00ab
	Novobiocin biosynthesis	0.07 + 0.01b	0.11 + 0.01a	0.11 + 0.00a	0.10 + 0.00a
	Phenazine biosynthesis	0.06 + 0.00a	0.05 + 0.00b	0.05 + 0.00ab	0.05 + 0.01b
	Staurosporine biosynthesis	0.00 + 0.00b	0.01 + 0.00ab	0.02 + 0.01ab	0.02 + 0.01a
	Streptomycin biosynthesis	0.28 + 0.01a	0.29 + 0.00a	0.26 + 0.01a	0.23 + 0.00b
	Tropane, piperidine and pyridine alkaloid biosynthesis	0.06 + 0.01b	0.11 + 0.01a	0.10 + 0.00a	0.09 + 0.00a
Carbohydrate metabolism	Amino sugar and nucleotide sugar metabolism	0.95 + 0.02a	0.82 + 0.03b	0.8 + 0.02b	0.76 + 0.05b
	Ascorbate and aldarate metabolism	0.10 + 0.02b	0.26 + 0.05a	0.20 + 0.00ab	0.21 + 0.02a
	Butanoate metabolism	0.50 + 0.03b	0.69 + 0.05a	0.78 + 0.00a	0.77 + 0.02a
	Citrate cycle (TCA cycle)	0.57 + 0.02b	0.56 + 0.01b	0.64 + 0.01a	0.59 + 0.02ab
	Glycolysis/Gluconeogenesis	0.97 + 0.01a	0.82 + 0.04bc	0.88 + 0.01b	0.80 + 0.02c
	Glyoxylate and dicarboxylate metabolism	0.87 + 0.02c	1.05 + 0.01b	1.11 + 0.02ab	1.19 + 0.06a
	Pentose phosphate pathway	0.64 + 0.01a	0.62 + 0.02ab	0.58 + 0.01b	0.59 + 0.02b
	Propanoate metabolism	0.61 + 0.03b	0.66 + 0.01b	0.74 + 0.01a	0.72 + 0.01a
	Pyruvate metabolism	1.08 + 0.01a	0.97 + 0.02c	1.03 + 0.01ab	1.01 + 0.01bc
	Starch and sucrose metabolism	0.68 + 0.03a	0.5 + 0.06b	0.56 + 0.04ab	0.57 + 0.03ab
Global and overview maps	2-Oxocarboxylic acid metabolism	0.74 + 0.01a	0.74 + 0.02a	0.73 + 0.03a	0.67 + 0.01b
	Biosynthesis of amino acids	3.42 + 0.04a	3.00 + 0.09bc	3.10 + 0.06b	2.88 + 0.02c
	Biosynthesis of secondary metabolites	8.82 + 0.10a	7.75 + 0.27b	8.12 + 0.09b	7.75 + 0.07b
	Degradation of aromatic compounds	0.22 + 0.02a	0.39 + 0.05b	0.37 + 0.00b	0.40 + 0.06b
	Fatty acid metabolism	0.69 + 0.03b	0.76 + 0.04ab	0.85 + 0.03a	0.77 + 0.02b
	Metabolic pathways	19.05 + 0.23a	17.77 + 0.33b	17.89 + 0.03b	17.85 + 0.04b
	Microbial metabolism in diverse environments	4.77 + 0.11b	5.21 + 0.09a	5.31 + 0.06a	5.42 + 0.04a
Glycan biosynthesis and metabolism	Arabinogalactan biosynthesis-Mycobacterium	0.04 + 0.00a	0.01 + 0.00bc	0.02 + 0.00b	0.01 + 0.00c
	Glycosaminoglycan degradation	0.02 + 0.00b	0.03 + 0.00a	0.04 + 0.00a	0.04 + 0.00a
	Glycosphingolipid biosynthesis-ganglio series	0.00 + 0.00b	0.01 + 0.00ab	0.01 + 0.00ab	0.01 + 0.00a
	Lipoarabinomannan (LAM) biosynthesis	0.01 + 0.00b	0.01 + 0.00b	0.03 + 0.00a	0.01 + 0.00b
	Lipopolysaccharide biosynthesis	0.25 + 0.00b	0.37 + 0.04a	0.32 + 0.04ab	0.3 + 0.03ab
	N-Glycan biosynthesis	0.01 + 0.00b	0.01 + 0.00b	0.02 + 0.00a	0.01 + 0.00b
	Other glycan degradation	0.01 + 0.00b	0.03 + 0.01ab	0.05 + 0.01a	0.05 + 0.01a
	Peptidoglycan biosynthesis	0.66 + 0.01a	0.52 + 0.03b	0.54 + 0.01b	0.5 + 0.02b
	Various types of N-glycan biosynthesis	0.00 + 0.00b	0.01 + 0.00ab	0.01 + 0.00a	0.01 + 0.00ab
Lipid metabolism	Arachidonic acid metabolism	0.01 + 0.00b	0.03 + 0.00a	0.03 + 0.00a	0.04 + 0.00a
	Ether lipid metabolism	0.01 + 0.00b	0.02 + 0.00ab	0.03 + 0.00a	0.03 + 0.01a
	Fatty acid biosynthesis	0.52 + 0.01a	0.5 + 0.01ab	0.53 + 0.02a	0.47 + 0.01b
	Fatty acid degradation	0.31 + 0.04b	0.45 + 0.04a	0.56 + 0.01a	0.51 + 0.04a
	Glycerolipid metabolism	0.33 + 0.00a	0.27 + 0.01b	0.27 + 0.01b	0.25 + 0.02b
	Glycerophospholipid metabolism	0.35 + 0.02c	0.43 + 0.01b	0.46 + 0.00ab	0.48 + 0.01a
	Primary bile acid biosynthesis	0.01 + 0.00ab	0.00 + 0.00b	0.01 + 0.00a	0.01 + 0.00ab
	Secondary bile acid biosynthesis	0.03 + 0.00a	0.03 + 0.00a	0.03 + 0.00ab	0.02 + 0.00b
	Sphingolipid metabolism	0.02 + 0.00c	0.03 + 0.01b	0.05 + 0.00ab	0.05 + 0.00a
	Steroid hormone biosynthesis	0.00 + 0.00c	0.01 + 0.00bc	0.02 + 0.00a	0.01 + 0.00ab
	Synthesis and degradation of ketone bodies	0.08 + 0.02b	0.18 + 0.03a	0.19 + 0.00a	0.19 + 0.01a
Metabolism of cofactors and vitamins	Folate biosynthesis	0.60 + 0.01a	0.54 + 0.02b	0.55 + 0.01b	0.54 + 0.00b
	Lipoic acid metabolism	0.12 + 0.01a	0.06 + 0.01b	0.06 + 0.00b	0.05 + 0.00b
	Nicotinate and nicotinamide metabolism	0.54 + 0.01a	0.47 + 0.01b	0.46 + 0.00bc	0.44 + 0.01c
	One carbon pool by folate	0.39 + 0.01a	0.32 + 0.01bc	0.34 + 0.01b	0.30 + 0.01c
	Porphyrin and chlorophyll metabolism	1.19 + 0.11a	0.85 + 0.09b	0.78 + 0.01b	0.84 + 0.1b
	Thiamine metabolism	0.38 + 0.01a	0.29 + 0.02b	0.3 + 0.01b	0.29 + 0.01b
	Ubiquinone and other terpenoid-quinone biosynthesis	0.40 + 0.02a	0.30 + 0.01b	0.25 + 0.01bc	0.22 + 0.03c
	D-Alanine metabolism	0.10 + 0.00a	0.09 + 0.01b	0.08 + 0.00b	0.08 + 0.00b
	D-Arginine and D-ornithine metabolism	0.01 + 0.00b	0.02 + 0.00a	0.02 + 0.00a	0.02 + 0.00a
	D-Glutamine and D-glutamate metabolism	0.15 + 0.01a	0.12 + 0.01b	0.10 + 0.00b	0.10 + 0.00b
	Glutathione metabolism	0.49 + 0.02b	0.54 + 0.01ab	0.50 + 0.02b	0.6 + 0.02a
	Phosphonate and phosphinate metabolism	0.06 + 0.01c	0.08 + 0.00c	0.10 + 0.00b	0.13 + 0.01a
	Selenocompound metabolism	0.29 + 0.01a	0.23 + 0.01b	0.3 + 0.01a	0.27 + 0.01ab
	beta-Alanine metabolism	0.19 + 0.02b	0.26 + 0.03a	0.27 + 0.01a	0.25 + 0.02ab
	Biosynthesis of vancomycin group antibiotics	0.04 + 0.00ab	0.05 + 0.01a	0.04 + 0.00ab	0.03 + 0.00b
	Geraniol degradation	0.05 + 0.01b	0.11 + 0.02a	0.12 + 0.00a	0.10 + 0.01a
	Insect hormone biosynthesis	0.03 + 0.01b	0.06 + 0.01ab	0.06 + 0.00a	0.05 + 0.01ab
	Limonene and pinene degradation	0.06 + 0.01b	0.11 + 0.02a	0.13 + 0.01a	0.11 + 0.01a
	Nonribosomal peptide structures	0.03 + 0.00a	0.02 + 0.00b	0.01 + 0.00b	0.01 + 0.00b
	Polyketide sugar unit biosynthesis	0.13 + 0.01ab	0.15 + 0.01a	0.13 + 0.01bc	0.11 + 0.00c
	Sesquiterpenoid and triterpenoid biosynthesis	0.05 + 0.01a	0.01 + 0.01b	0.01 + 0.00b	0.02 + 0.00b
	Terpenoid backbone biosynthesis	0.42 + 0.00a	0.37 + 0.01b	0.42 + 0.01a	0.39 + 0.01ab
	Zeatin biosynthesis	0.03 + 0.00a	0.02 + 0.00b	0.03 + 0.00b	0.02 + 0.00b

Different letters after the data indicate a significant difference level of 5%. Values represent mean relative abundance within sample (%) ± standard error (n=3).

## 4 Discussion

The ripening process of medicinal plants represents a sophisticated interplay between host plant physiology and its associated endophytic microbiota (Trivedi et al., 2020; Xu et al., 2023). Recent ecological theories emphasize that such host-microbe partnerships are fundamental to plant fitness and metabolic capacity, conceptualized under the holobiont framework (Toju et al., 2018; Spooren et al., 2024). This study provides a comprehensive, multi-omics perspective on the dynamic co-evolution of the endophytic microbiome and the metabolome during the fruit development of *R. chingii*, a plant of significant medicinal and economic importance. The findings strongly suggest that the distinct metabolic profiles defining the quality of unripe (medicinal) and ripe (edible) fruits are closely linked to stage specific shifts in the endophytic community (Toju et al., 2018; Kapoor et al., 2022).

A central finding of our study is the pronounced decline in the concentrations of key secondary metabolites, including phenolic acids, flavonoids, ellagic acid, and several flavonoid glycosides, as the fruit matures. This trend is consistent with ripening, associated metabolic shifts observed in other rosaceous fruits (Ma et al., 2024), where a reallocation of resources from defense, related secondary metabolism towards primary metabolism and attraction traits (e.g., sugar accumulation) often occurs (Zhang et al., 2025; Jia et al., 2024; Aaby et al., 2012). Notably, a combined transcriptomic and metabolomic study on *R. chingii* fruit development revealed that the biosynthesis of phenolic acids and flavonols is initiated early in fruit set and subsequently declines during ripening, further supporting the metabolic trade-off observed in our system (Chen et al., 2021). This metabolic trade-off is a common phenomenon in fleshy fruits, driven by evolutionary pressures to balance defense with dispersal (Droby and Wisniewski, 2018; Chi et al., 2023). The observations align with metabolomic studies in other medicinal berries, such as *Lycium barbarum*, where a similar decline in specific glycosides was linked to fruit maturation, underscoring a potential conserved pattern in medicinally active compounds (Zhao et al., 2020). The transcriptional downregulation of pivotal genes in the phenylpropanoid pathway during the later stages of fruit development is a well-documented phenomenon that likely underlies this observed metabolic pattern (Cao et al., 2024). Emerging evidence suggests that the plant microbiome can influence host gene expression, potentially including those involved in secondary metabolism (Chen et al., 2022; Li Y. C. et al., 2021). For example, a study on *Ginkgo biloba* revealed that endophytic bacteria could modulate the expression of key genes in the flavonoid pathway, suggesting a direct mechanistic link between microbial presence and host metabolic output (Fu et al., 2022). Although not explicitly examined in the present study, such microbiome-mediated regulation may represent an additional layer of control over the metabolic shifts observed during *R. chingii* fruit maturation. Consequently, the potent antioxidant capacity, predominantly attributed to these phenolic compounds (Chen Y. et al., 2020; Zhang M. Q. et al., 2023), diminished significantly in the ripe red fruits (RYR), as evidenced by the sharply increased EC_50_ values in the DPPH assay. This inverse relationship between ripeness and bioactivity underpins the traditional practice of harvesting unripe *R. chingii* fruits for medicinal use. Conversely, the accumulation of polysaccharides in RYR fruits aligns with their role as a functional food, potentially contributing to texture and nutritional value (Zhang et al., 2015).

High-throughput sequencing revealed that the fruit of *R. chingii* hosts a diverse and dynamic endophytic microbiome, the structure of which is strongly influenced by fruit ripening stage. The alpha-diversity of bacterial communities exhibited a unimodal pattern, peaking at the intermediate yellow stage (RYY). Bacterial alpha-diversity followed a unimodal pattern, reaching its peak at the intermediate yellow stage (RYY). This trend suggests the existence of a transitional phase during which fruit internal conditions favor maximal taxonomic richness, prior to becoming more selective in later maturation; a phenomenon also reported in the berry microbiomes of grape (Ding et al., 2021) and raspberry (Sangiorgio et al., 2022). Hump-shaped diversity pattern during ecological succession is consistent with theoretical predictions and has been empirically documented in the leaf microbiomes of perennial plants, pointing to shared assembly principles across plant compartments (Shoemaker et al., 2025). NMDS ordination, supported by PERMANOVA results, further confirmed that ripening stage serves as a key determinant of beta-diversity. The early ripening stage (RYL) formed a distinct cluster, indicating the establishment of a unique pioneer microbial community in unripe fruits, likely shaped by their firm texture, high acidity, and abundance of defensive metabolites (Malacrinò et al., 2022). Plant tissue physicochemical traits are recognized as critical filters in microbial community assembly (Coleman-Derr et al., 2016; Trivedi et al., 2020; Allard and Micallef, 2019), and the high phenolic content in immature fruits in particular may impose strong selective pressure, enriching for microorganisms equipped with phenolic tolerance or degradation abilities: a pattern similarly observed in the rhizosphere of other phenolic rich plants (Bao et al., 2022; Hurtado-Navarro et al., 2025).

Taxonomic analysis revealed a clear successional pattern characterized by significant species turnover in the endophytic microbiome, which transitioned from a community dominated by *Cyanobacteriota* in unripe fruits to one increasingly governed by *Pseudomonadota* in later developmental stages. This successional dynamic aligns with the conceptual framework of ecological succession, where the changing host environment drives the replacement of microbial taxa over time. This shift appears to reflect fundamental changes in the fruit’s internal environment, including the degradation of chloroplasts and the rising availability of soluble sugars as ripening progresses. Accordingly, at the green fruit stage, we observed the enrichment of genera such as *Massilia* and *Pseudomonas*, both recognized for their capacity to degrade plant cell wall components, suggesting an active role in early maturation (Zhang G. H. et al., 2023; Chen T. et al., 2020). Conversely, as fruits enter the red stage, a pronounced increase in *Methylobacterium* becomes notable. This genus, known for its methylotrophic metabolism, can utilize methanol from pectin demethylation, positioning it as a key participant in the later stages of cell wall disassembly (Grossi et al., 2024). This successional trajectory underscores the principle of metabolic niche partitioning within the endospheric microenvironment (Lindow and Brandl, 2003; Compant et al., 2019), a pattern supported by similar findings in mango (Bill et al., 2021). In contrast, the fungal community exhibited greater structural stability, consistently dominated by the generalist genus *Cladosporium*. Nevertheless, subtle compositional shifts, such as variations in yeasts like *Metschnikowia* and *Starmerella*, hint at underlying functional adjustments. This pattern of fungal stability coupled with bacterial turnover has been documented in other fruit systems, suggesting divergent ecological strategies between these two microbial kingdoms (Sun et al., 2021). The persistent dominance of *Cladosporium* may be explained by its broad enzymatic capabilities and pronounced stress tolerance, traits frequently associated with core fungal endophytes (Junker and Keller, 2015).

The most significant contribution of this study resides in the construction of a correlation network that explicitly links specific microbial taxa with the accumulation of valuable fruit metabolites. The positive correlations observed between several bacterial genera, such as *Brevundimonas, Modestobacter, Cellulomonas* and *Geodermatophilus* and the content of flavonoids and phenolic acids, suggest their potential involvement in enhancing or stabilizing these bioactive compounds. This functional inference is biologically plausible, as endophytic Actinobacteria are widely recognized for their versatile enzymatic systems capable of modifying plant, derived molecules, alongside their ability to elicit systemic resistance in the host, a process that often upregulates defense-related metabolic pathways (Aamir et al., 2020; Zhang et al., 2018). Notably, several studies have demonstrated that endophytic Actinobacteria can produce phytohormone analogs or activate key host signaling pathways, such as those mediated by jasmonic acid and salicylic acid, which serve as central regulators of plant secondary metabolism (Passari and Upadhyaya, 2017; Ketehouli et al., 2025). The ecological relevance of these interactions is further underscored by a recent meta-analysis, which identified Actinobacteria as one of the bacterial taxa most consistently associated with enhanced secondary metabolite accumulation across a wide range of plant species (Aamir et al., 2020). Similar mechanisms have been reported in other beneficial bacteria; for example, certain *Bacillus* species are known to stimulate flavonoid biosynthesis in medicinal plants (Ji et al., 2025; Mierziak et al., 2014). In this context, the correlation between *Bradyrhizobium*, *Actinomycetospora* and rutin is of particular interest, given that flavonoids not only function as defensive end products but also act as key signaling molecules in plant-microbe communication.

In addition to the specific correlations highlighted, the correlation analysis also revealed a broad pattern of non-significant associations between the majority of microbial genera and fruit metabolites. The prevalence of these non-significant relationships suggests that the influence of the endophytic community on metabolite composition may not be universally direct or strong, but rather concentrated in specific keystone taxa, as observed with genera like *Brevundimonas* and *Starmerella*. This could be attributed to several factors: the metabolic activity of many endophytes might be constitutive and not directly linked to the specific secondary metabolites measured; functional redundancy within the microbial community could diffuse observable correlations for individual members; or the impact of the microbiome may be more systemic, influencing the host’s metabolic pathways indirectly rather than through the direct production of these compounds. Therefore, the observed metabolite profile is likely the result of complex multi-trophic interactions within the fruit, alongside strong host genetic and developmental regulation.

Among the fungal community, the positive associations observed between non-Saccharomyces yeasts, particularly *Udeniomyces*, *Metschnikowia*, *Starmerella* and phenolic compounds suggest a potential synergistic relationship. These yeasts, which are commonly present in fruit-related niches, have been reported to positively modulate the phenolic profile during fermentation, largely due to their capacity to secrete enzymes such as β-glucosidases, which catalyze the transformation of phenolic glycosides into their active forms (Zhang et al., 2023; Portu et al., 2016). Beyond their established role in fermentation, their presence as endophytes may support host fitness through mechanisms such as detoxification of antimicrobial compounds or by providing growth-stimulating signals, thereby indirectly influencing metabolic fluxes within plant tissues (Freimoser et al., 2019). The ability of these yeasts to modify the phenolic composition is not only relevant to fruit defense during development but may also influence postharvest quality, as evidenced by their successful application as biocontrol agents against fruit pathogens (Spadaro and Droby, 2016). Therefore, the abundance of *Udeniomyces*, *Metschnikowia* and *Starmerella* in the fruit endosphere may reflect a functional contribution to the modification or stabilization of the host phenolic pool.

Functional predictions provided a deeper, albeit inferred, layer of understanding of microbial metabolic dynamics during fruit maturation. PICRUSt2 analysis indicated a distinct shift in bacterial metabolic potential, transitioning from fundamental processes such as amino acid biosynthesis in unripe fruit to more specialized functions, including glutathione and sphingolipid metabolism in ripe fruit. This transition likely reflects a community, wide adaptation to ripening, related oxidative stress and a potential role in membrane remodeling during tissue softening. The predicted enhancement of sphingolipid metabolism is particularly suggestive of bacterial involvement in membrane dynamics, as sphingolipids are crucial structural and signaling lipids known to mediate membrane integrity, programmed cell death, and senescence processes in plants (Liu et al., 2021). Concurrently, the upregulation of glutathione metabolism pathways points to a microbial response to oxidative stress, given glutathione’s well-established role as a master antioxidant regulator in redox homeostasis during plant development and stress (Miret and Munné-Bosch, 2014). Such a metabolic reconfiguration is consistent with the emerging paradigm where plant-associated microbiomes actively contribute to host stress mitigation, including coping with the oxidative burst associated with ripening (Ali et al., 2022). The predicted enhancement of glutathione metabolism is particularly noteworthy, given glutathione’s central function in maintaining cellular redox homeostasis amid the oxidative burst characteristic of ripening and senescence (Decros et al., 2019). The plausibility of these predictions is further supported by metatranscriptomic studies on fruit-surface microbiomes, which have similarly documented the upregulation of microbial genes involved in oxidative stress response as fruits enter the ripening stage (Sharma et al., 2022). Complementing these bacterial insights, FUNGuild analysis revealed an increase in saprotrophic fungi in red stage fruit (RYR), illustrating a classic ecological succession pattern wherein decomposers thrive as soluble sugars accumulate and tissue integrity declines, a hallmark of ripe fruit microbial communities. This progression toward saprotrophy aligns with the broader concept of the fruit microbiome shifting from a biotrophic to a necrotrophic phase as the organ senesces, thereby facilitating seed dispersal and nutrient recycling (Droby and Wisniewski, 2018).

## 5 Conclusion

In conclusion, our data support a model wherein the maturation of *R. chingii* fruit is a coordinated tripartite process involving the host developmental program, the dynamic succession of the endophytic microbiome, and resultant metabolite shifts. Key findings reveal a significant decline in bioactive metabolites (ellagic acid, flavonoids, and phenolic acids) and antioxidant capacity alongside a substantial microbial community restructuring during ripening. Crucially, specific bacterial (e.g., *Geodermatophilus*) and fungal (e.g., *Metschnikowia*) taxa, identified via robust correlation analysis, are proposed as potential active contributors to the fruit’s chemical identity. This opens up possibilities for microbiome based strategies to enhance the quality of this medicinal plant. Future research should move beyond correlation to causation by isolating these key microbes and validating their functions through re-inoculation experiments and Synthetic Community (SynCom) approaches. Integrating meta transcriptomics will be essential to unravel the molecular mechanisms of these interactions, thereby advancing the sustainable cultivation and therapeutic application of *R. chingii*.

## Data Availability

The endophytic bacteria and fungi raw RNA-seq datasets can be found in the NCBI SRA under the project number: PRJNA1303412. https://www.ncbi.nlm.nih.gov/sra/PRJNA1303412.
