# Supplementary material for: Endophyte community shifts in Rubus chingii during fruit ripening are associated with key metabolites
Source: Front Plant Sci. 2025 Dec 19;16:1727436. doi: 10.3389/fpls.2025.1727436 (PMC12757375; doi:10.3389/fpls.2025.1727436)
Supplement: Supplementary file 1 [file DataSheet1.docx]

Supplementary Material

# Supplementary Data

Supplementary Material should be uploaded separately on submission. Please include any supplementary data, figures and/or tables.

Supplementary material is not typeset so please ensure that all information is clearly presented, the appropriate caption is included in the file and not in the manuscript, and that the style conforms to the rest of the article.

# Supplementary Figures and Tables

For more information on Supplementary Material and for details on the different file types accepted, please see [here](https://www.frontiersin.org/guidelines/author-guidelines#supplementary-material).

## Supplementary Figures


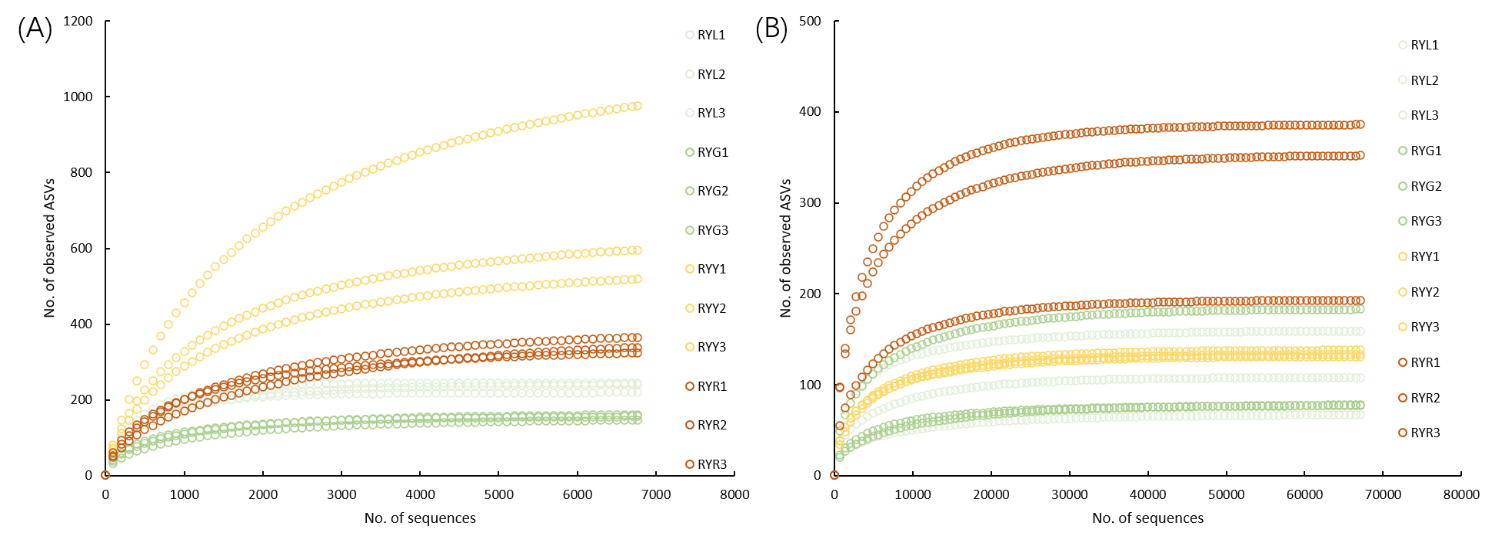


**Supplementary Figure 1.** Rarefaction curves of both endophytic bacteria (A) and fungi (B) in fruit of *R. chingii* from four harvest stages, depicting the effects of 3% dissimilarity on the number of ASVs identified. RYL (light green), RYG (green), RYY (yellow), RYR (red). The rarefaction curves for RYY and RYR groups show greater dispersion, reflecting the inherent biological variability in microbial richness among fruit samples at these later ripening stages."


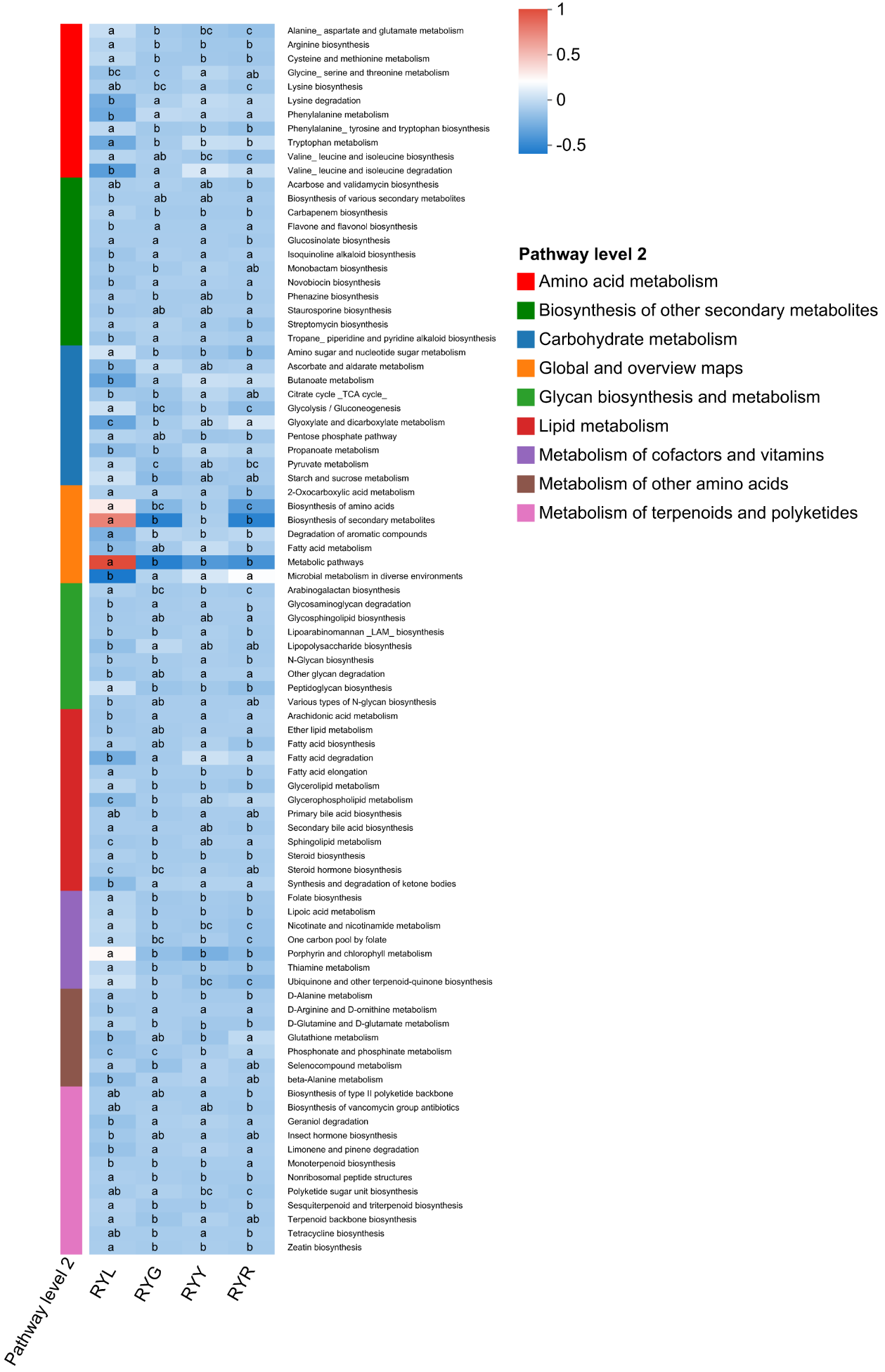


**Supplementary Figure 2.** Predicted function of the bacterial community found in *R. chingii* fruits based on KEGG pathway level 2 with significant difference among the four harvest stages. Different letters after the data indicate a significant difference level of 5%.


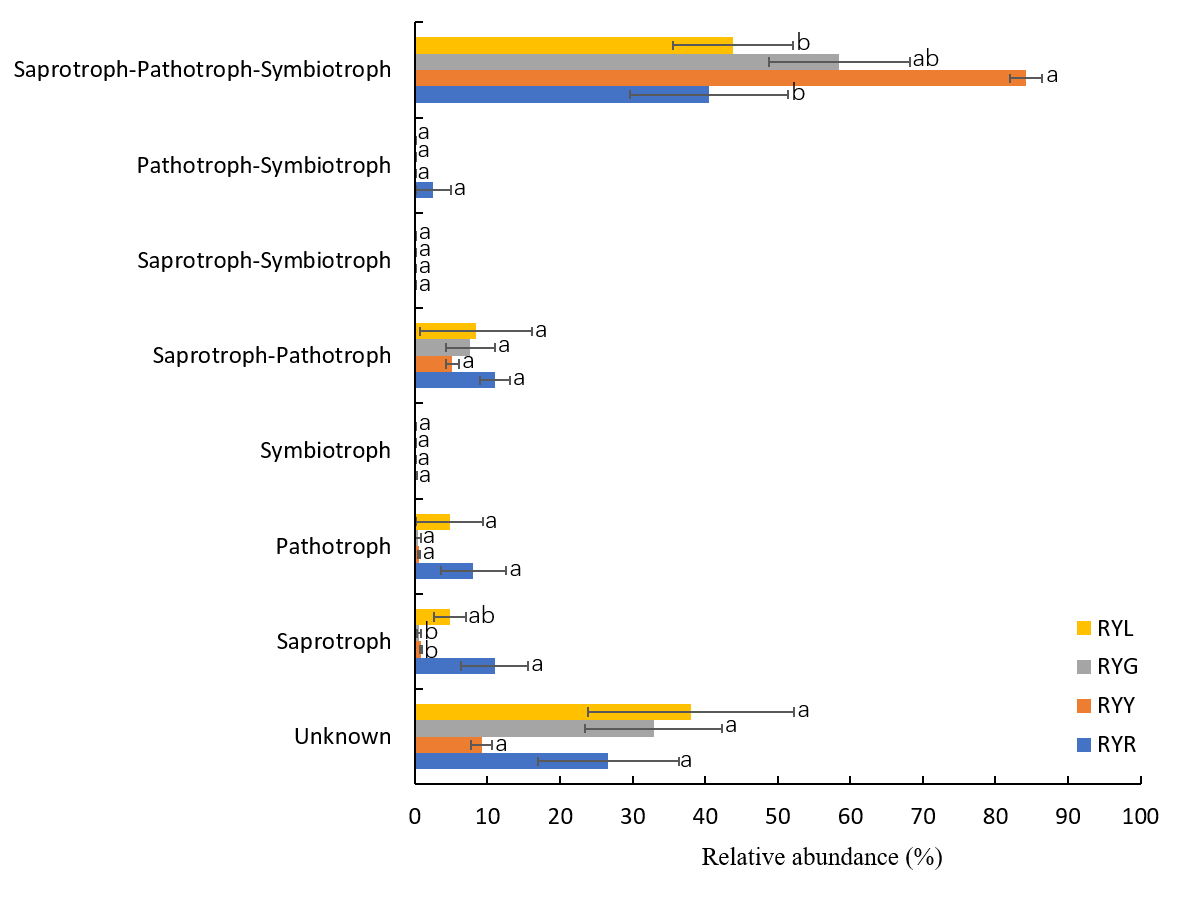


**Supplementary Figure 3.** **Predicted functional profiles of the endophytic bacterial community.**
The stacked bar chart shows the relative abundance of level-1 KEGG pathways across the four harvest stages (RYL, RYG, RYY, RYR) of *R. chingii* fruit. Functional categories are color-coded as indicated. Different lowercase letters above the bars indicate statistically significant differences (P < 0.05) in the overall functional profile between stages, as determined by PERMANOVA.
